# Genes underlying delayed puberty

**DOI:** 10.1016/j.mce.2018.05.001

**Published:** 2018-11-15

**Authors:** S.R. Howard

**Affiliations:** Centre for Endocrinology, William Harvey Research Institute, Barts and the London School of Medicine and Dentistry, Queen Mary University of London, London, EC1M 6BQ, UK

**Keywords:** Puberty, Pubertal timing, Delayed puberty, Self-limited delayed puberty, Constitutional delay, Puberty genetics, IGSF10

## Abstract

The genetic control of pubertal timing has been a field of active investigation for the last decade, but remains a fascinating and mysterious conundrum. Self-limited delayed puberty (DP), also known as constitutional delay of growth and puberty, represents the extreme end of normal pubertal timing, and is the commonest cause of DP in both boys and girls. Familial self-limited DP has a clear genetic basis. It is a highly heritable condition, which often segregates in an autosomal dominant pattern (with or without complete penetrance) in the majority of families. However, the underlying neuroendocrine pathophysiology and genetic regulation has been largely unknown. Very recently novel gene discoveries from next generation sequencing studies have provided insights into the genetic mutations that lead to familial DP. Further understanding has come from sequencing genes known to cause GnRH deficiency, next generation sequencing studies in patients with early puberty, and from large-scale genome wide association studies in the general population. Results of these studies suggest that the genetic basis of DP is likely to be highly heterogeneous. Abnormalities of GnRH neuronal development, function, and its downstream pathways, metabolic and energy homeostatic derangements, and transcriptional regulation of the hypothalamic-pituitary-gonadal axis may all lead to DP. This variety of different pathogenic mechanisms affecting the release of the puberty ‘brake’ may take place in several age windows between fetal life and puberty.

## Highlights

• Pubertal timing is strongly determined by genetics, but also by factors such as BMI and psychosocial environment.
• Self-limited delayed puberty (DP) is the commonest cause of DP, but only a small number of genetic causes are known.
• Other rarer genetic causes of DP include mutations in GnRH deficiency genes and primary hypogonadism.
• Gene discovery in DP is expanding via next generation sequencing and genome wide association approaches.

## 1 Introduction

The development of the hypothalamic-pituitary-gonadal (HPG) axis is remarkable, with GnRH neurons originating in metazoan embryos outside of the central nervous system. These neurons then undergo a coordinated and timely migration alongside olfactory and terminal axons during fetal life. Immature GnRH precursor neurons are first detectable in the olfactory placode in the nose from an early embryological stage, and then begin a complex journey towards the hypothalamus (Cariboni et al., 2007; Wray et al., 1989). The HPG axis is measurably active in fetal and again in early infant life, during the so-called ‘mini-puberty’, but thereafter becomes largely dormant between the age of one and eight-to-nine years (Beate et al., 2012). Development of the clinical features of puberty is initiated by the reactivation of the HPG axis after this relative quiescence during childhood. However, the nature of the puberty ‘brake’ that acts on the axis after the mini-puberty, and how this puberty brake is released – and the how the timing of release is controlled – to allow for puberty onset, is not well understood.

Despite the demonstrated importance of environmental factors such as body mass, psychosocial stressors and endocrine disrupting chemicals (EDCs) (de Muinich Keizer and Mul, 2001), genetic influence on the timing of puberty is clearly fundamental. Whilst the timing of pubertal onset varies within and between different populations, it is a highly heritable trait. Twin studies demonstrate that the timing of sexual maturation is highly correlated between highly related individuals, suggesting strong genetic determinants (Wehkalampi et al., 2008a). Previous studies estimate that 60–80% of the variation in pubertal onset is under genetic regulation (Gajdos et al., 2009; Parent et al., 2003). However, despite this strong heritability, the key genetic factors that determine human pubertal timing in the normal population and in cases of disturbed pubertal timing remain mostly unknown (Palmert and Dunkel, 2012a).

### 1.1 Causes of delayed puberty

The pathogenesis of delayed puberty (DP) encompasses several conditions, but is most commonly due to self-limited DP. There are two main groups of differential diagnosis of DP (Table 1): hypogonadotropic hypogonadism (HH) due to either functional or primary GnRH deficiency, and disorders causing primary hypogonadism (Palmert and Dunkel, 2012a; Sedlmeyer and Palmert, 2002), although up to 30 different aetiologies underlying DP have been identified (Varimo et al., 2017).

**Table 1:** Differential diagnoses of self-limited delayed puberty.

	Hypergonadotropic Hypogonadism	Primary Hypogonadotropic Hypogonadism	Functional Hypogonadotropic Hypogonadism
Common Causes:	Klinefelter Syndrome	Isolated Hypogonadotropic	Inflammatory Bowel Disease
	Gonadal dysgenesis including Turner's syndrome	Hypogonadism	Coeliac Disease
	Chemotherapy/Radiation Therapy	Kallmann syndrome	Anorexia Nervosa
		Combined Pituitary Hormone	Hypothyroidism
		Deficiency	Excessive Exercise
		Chemotherapy/Radiation Therapy	
		CNS Tumours/Infiltrative Diseases	

Table modified and reprinted with permission from Palmert MR, Dunkel L. Clinical practice. Delayed puberty. N Engl J Med 2012; 366:443–53 (Palmert and Dunkel, 2012b).

Self-limited DP, also known as constitutional delay of growth and puberty (CDGP), represents the commonest cause of DP in both sexes. Up to 83% of boys and 30% of girls with pubertal delay have self-limited DP (Sedlmeyer and Palmert, 2002; Varimo et al., 2017; Abitbol et al., 2016; Lawaetz et al., 2015). The underlying reasons for this gender difference are not clear. HH due to GnRH deficiency, such as in Kallmann syndrome (KS) and idiopathic HH, is also seen more commonly in men than in women. In contrast to DP and HH, precocious onset of puberty is approximately five times more common in girls than boys. The prevalence of central precocious puberty (CPP) in girls was found to be 0.2%, but only 0.05% in boys, over a nine year period in one European series (Teilmann et al., 2005). However, an underlying abnormality is found far more commonly in girls with DP, and in boys with CPP, suggesting that many cases of male DP and female CPP may represent the end of the normal spectrum without underlying pathology.

There are fundamental differences between the two sexes in the dynamics of the reactivation of the gonadotropic axis. The biological reactivation of the gonadotropic axis occurs earlier in girls than boys. In boys, the secretion of testosterone increases shortly after the increase in the plasma concentration of luteinizing hormone (LH) and follicular stimulating hormone (FSH). In girls, estradiol increases together with increasing LH and FSH. It is unknown how the genetic differences between the sexes contribute to this sexual dimorphism. There is some evidence that the female HPG axis may be more sensitive to environmental factors such as increased fat mass than the male, such as in conditions of functional hypogonadism due to weight loss or excessive exercise, where women tend to be affected more than men.

Individuals with DP have age of pubertal onset outside of the statistical definition of normal pubertal timing, with the absence of testicular enlargement in boys or breast development in girls at an age that is 2–2.5 standard deviations (SD) later than the population mean (Palmert and Dunkel, 2012a). In addition, DP may also encompass older children with delayed pubertal progression, a diagnosis that is aided by the use of puberty normograms (Lawaetz et al., 2015). Self-limited DP may be associated with adverse health outcomes including short stature, reduced bone mineral density and compromised psychosocial health (Zhu and Chan, ).

Differential diagnosis between self-limited DP and HH in adolescents who present with DP is often difficult at the time of referral, as both conditions may present with effectively the same clinical and hormonal features. Whilst a variety of clinical and biochemical investigations are available in such patients, none can reliably distinguish between those patients who will spontaneously enter, and progress in a normal manner, through puberty, and those who will require significant short or long term medical management. As the understanding of the genetic basis of both self-limited and other causes of DP improves, it is likely that genetic testing will be able to help establish a definitive diagnosis in such cases.

### 1.2 Investigating the inheritance of self-limited delayed puberty

**Fig. 1:**
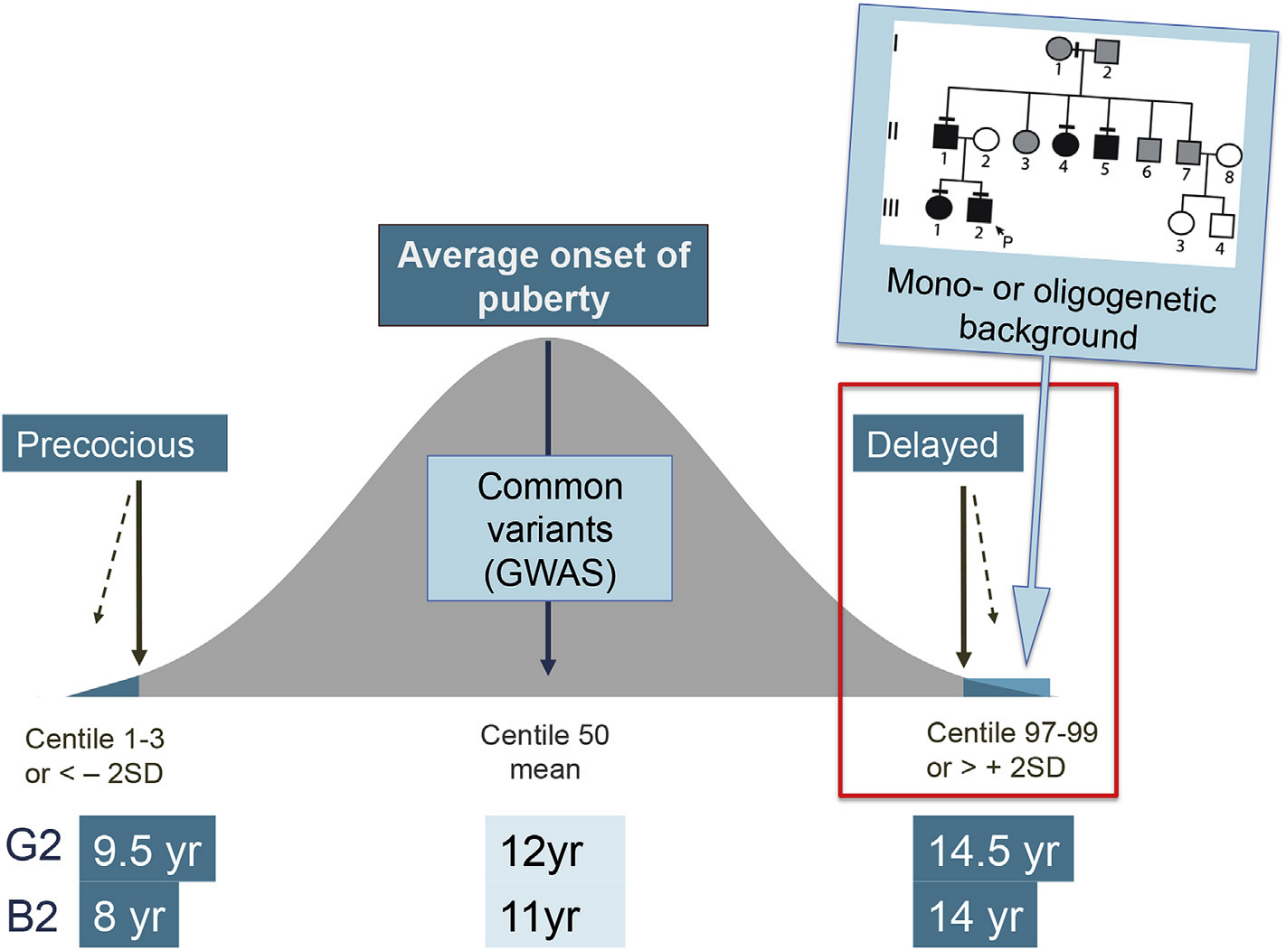
**– The Genetics of Pubertal Timing.** In the general population there is a near-normal distribution of the timing of pubertal onset, with the definitions of precocious and delayed being statistically determined ( ±2 standard deviations, SD). Cut-off ages for Tanner genital stage G2 (boys) and B2 (girls) defining precocious and delayed puberty are given (thick black lines represent 3rd and 97th centiles and dotted lines represent 1st and 99th centiles). Strategies to determine key genetic determinants in the timing of puberty include large genome wide association studies (GWAS) of age-at-menarche and voice breaking in the general population (common variants box), and identification of rare high-impact variants causing early, late or absent puberty in patients and their families. Patients with familial self-limited DP often display an autosomal dominant mode of inheritance, likely with a mono- or oligogenetic basis.

Self-limited DP segregates within families with complex patterns of inheritance including autosomal dominant, autosomal recessive, bilineal and X-linked (Sedlmeyer, 2002), although sporadic cases are also observed. The majority of families display an autosomal dominant pattern of inheritance (with or without complete penetrance) (Fig. 1) (Wehkalampi et al., 2008a; Sedlmeyer, 2002). 50–75% of subjects with self-limited DP have a family history of delayed pubertal onset (Sedlmeyer, 2002).

Some insights into the genetic mutations that lead to familial self-limited DP have come from sequencing genes known to cause GnRH deficiency, most recently via next generation sequencing. Linkage analysis and targeted sequencing strategies that have provided initial insights in this field (Cousminer et al., 2015; Wehkalampi et al., 2008b) have been mostly superseded by whole exome and genome sequencing strategies to identify novel candidate genes. Other candidates have been identified from large-scale genome wide association studies in the general population.

Analysis of self-limited DP families is complicated by the fact that this phenotype represents the tail of a normally distributed trait within the population, so it is expected that variants that govern the inheritance of this condition may also be present in the general population at a low level. Thus, the absence of these variants in population databases cannot be used as an exclusion criterion during filtering of sequencing data. Instead, a comparison of prevalence of such variants must be made to identify those that are enriched in patients compared to the general population. To date, in the majority of patients with DP the neuroendocrine pathophysiology and its genetic regulation remain unclear.

### 1.3 Novel genetics discoveries in self-limited delayed puberty

**Fig. 2:**
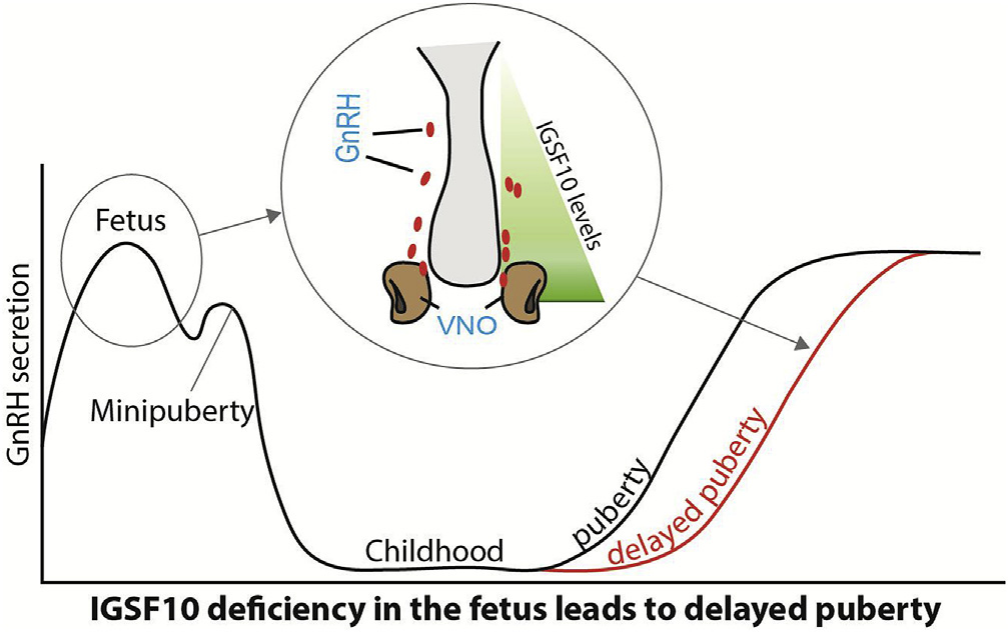
**– Schematic of the mechanism by which *IGSF10* mutations lead to DP.** Reduced levels of *IGSF10* expression during embryogenesis (represented by green triangle) in the corridor of nasal mesenchyme from the vomeronasal organ (VNO) to the olfactory bulbs (in a murine model) result in delayed migration of GnRH neurons (represented by red ovals) to the hypothalamus. This presents for the first time in adolescence as a phenotype of DP due to abnormalities of the GnRH neuronal network (grey arrows linking fetal pathogenesis to adolescent phenotype). (For interpretation of the references to colour in this figure legend, the reader is referred to the Web version of this article.)

Recently, whole exome and targeted resequencing methods have implicated two pathogenic mutations in *Immunoglobulin superfamily member 10 (IGSF10)* as the causal factor for late puberty in six unrelated families from a large Finnish cohort with familial DP (Howard et al., ). A further two rare variants of unknown significance were identified in four additional families from the cohort. Mutations in *IGSF10* appear to cause a dysregulation of GnRH neuronal migration during embryonic development (Fig. 2), which presents in adolescence as DP without previous constitutional delay in growth. An intact GnRH neurosecretory network is necessary for the correct temporal pacing of puberty. Pathogenic *IGSF10* mutations leading to disrupted IGSF10 signalling potentially result in reduced numbers or mis-timed arrival of GnRH neurons at the hypothalamus; producing a functional defect in the GnRH neuroendocrine network. With this impaired GnRH system there would follow an increased ‘threshold’ for the onset of puberty, with an ensuing delay in pubertal timing. *IGSF10* loss-of-function mutations were also discovered in patients with a hypothalamic amenorrhea-like phenotype. Although loss-of-function mutations in *IGSF10* were enriched in patients with HH, these mutations did not alone appear sufficient to cause the phenotype of full GnRH deficiency, in view of lack of complete segregation with trait. These findings represent a new fetal origin of self-limited DP, and reveal a potential shared pathophysiology between DP and other forms of functional hypogonadism.

Loss-of-function mutations in a member of the immunoglobulin superfamily, Immuno*globulin superfamily member 1 (IGSF1)*, have been identified in patients with X-linked central hypothyroidism (Sun et al., 2012). Notably, male patients with *IGSF1* mutations have a late increase in testosterone levels with a delayed pubertal growth spurt. However, pathogenic mutations in *IGSF1* have not been conclusively found in patients with isolated DP (Joustra et al., 2015).

### 1.4 Relevance of established GnRH deficiency genes to DP

**Fig. 3:**
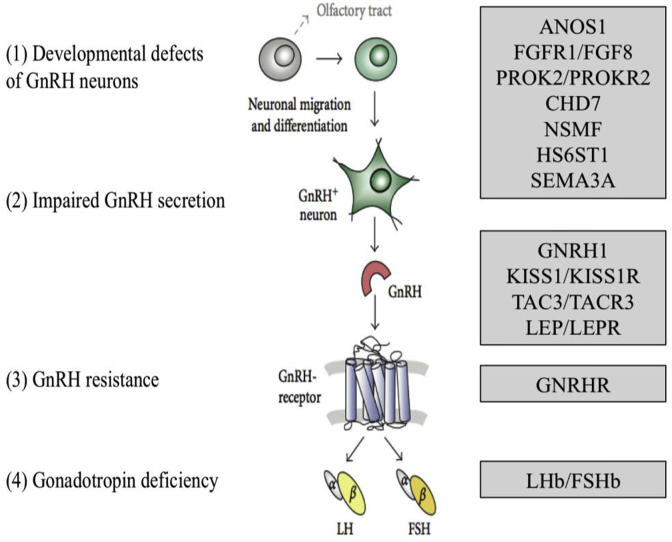
**– Mutations in single genes at many levels of the HPG axis can cause hypogonadotropic hypogonadism** (adapted from (Beate et al., 2012)).

**Fig. 4:**
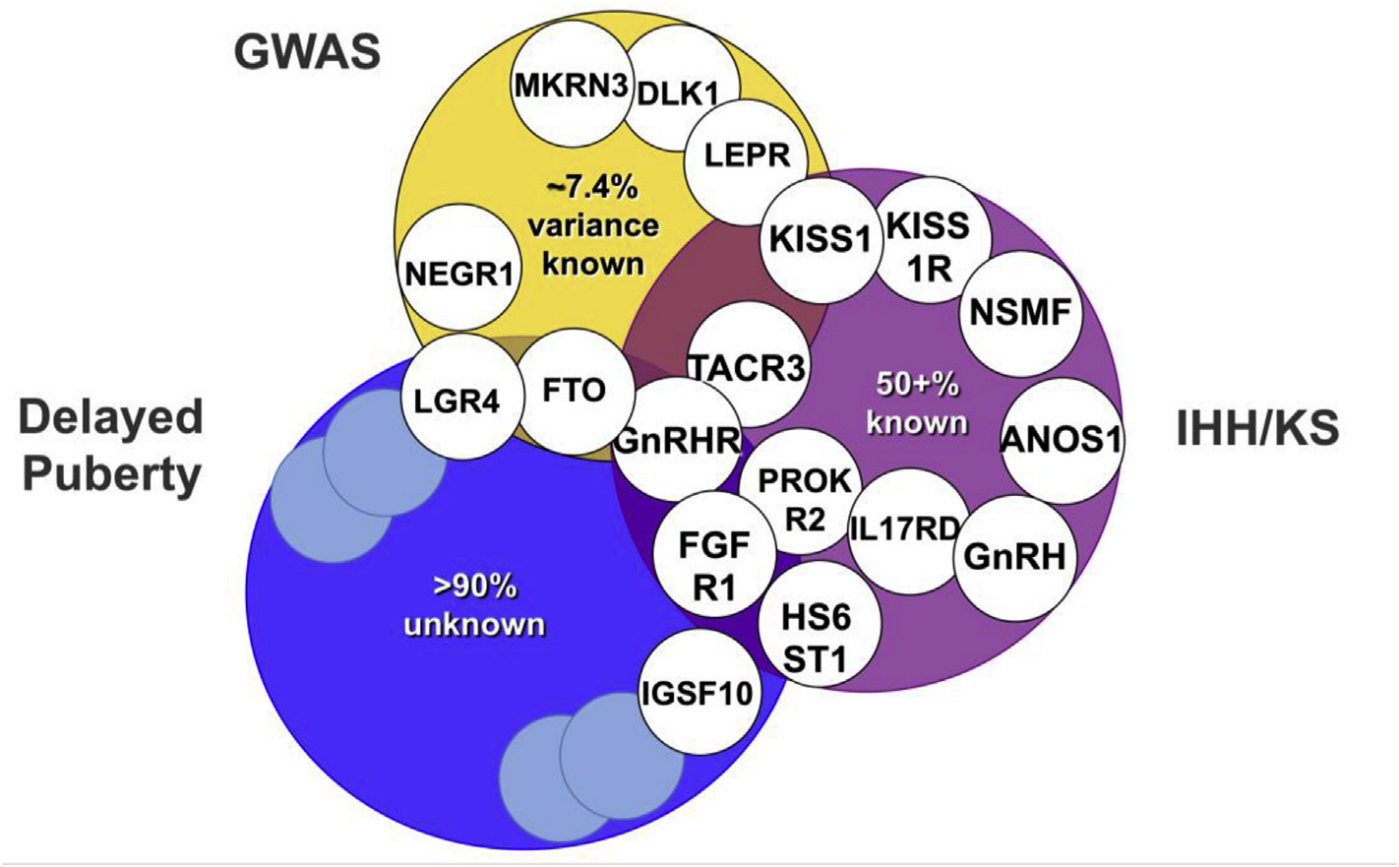
**Overlap between genetic regulation in the general population and extreme phenotypes**. Examples of genes implicated in timing of puberty from genome wide association studies in the general population (GWAS), conditions of GnRH deficiency such as idiopathic hypogonadotropic hypogonadism (IHH) and Kallmann Syndrome (KS), and self-limited delayed puberty. Pale blue unfilled circles represent as yet undiscovered genes. (For interpretation of the references to colour in this figure legend, the reader is referred to the Web version of this article.)

At the extreme end of the spectrum of DP are conditions of GnRH deficiency including congenital hypogonadotropic hypogonadism (CHH), with complete failure to enter puberty. The condition may be due to failure of development of GnRH neurons, lack of activation of GnRH secretion or disrupted GnRH signalling (Fig. 3). Because of different causes and incomplete penetrance, there is a wide spectrum of phenotypes, ranging from complete CHH with lack of pubertal development to a partial hypogonadism with an arrest of pubertal development, and reversible CHH in up to 20% of patients post treatment (Raivio et al., 2007; Pitteloud et al., 2007; Sidhoum et al., 2014). Despite recent advances, with over forty genes linked to this disorder identified, the pathophysiological basis of CHH in approximately 50% of individuals remains unclear (Fig. 4) (Beate et al., 2012).

In view of the possible overlap between the pathophysiology of DP and conditions of GnRH deficiency, a few studies have specifically examined the contribution of mutations in CHH genes to the phenotype of self-limited DP. Mutations in *Heparan Sulfate 6-O-Sulfotransferase 1 (HS6ST1)*, *Fibroblast Growth Factor Receptor 1 (FGFR1)* and newly in *Klotho Beta (KLB)* have been found in a small number of kindreds of CHH patients and their relatives with DP (Tornberg et al., 2011; Pitteloud et al., 2006; Xu et al., ). Variants in several HH genes including *Gonadotropin Releasing Hormone Receptor (GNRHR)*, *Tachykinin 3 (TAC3)* and its receptor *(TACR3)*, *Interleukin 17 Receptor D (IL17RD)* and *Semaphorin 3A (SEMA3A)* have been identified by whole exome sequencing in some cases of DP, including self-limited DP (Zhu et al., ). However, these variants have not been tested *in vitro* or *in vivo* for pathogenicity, or investigated for segregation with trait within pedigrees, and thus may be an over-estimation. Most recently, a comparative study of the frequency of mutations in 24 GnRH deficiency genes between probands with congenital HH and those with self-limited DP found a significantly higher proportion of mutations in the HH group (51% of HH probands vs 7% of DP probands, *p* = 7.6 × 10^−11^), with a higher proportion of oligogenicity in the HH group, suggesting distinct genetic profiles in these two conditions (Cassatella et al., 2018). Mutations in KS genes such as *Anosmin 1 (ANOS1)* and *NMDA Receptor Synaptonuclear Signaling And Neuronal Migration Factor (NSMF)* have not to date been identified in pedigrees with DP. Overall, the current picture indicates that the genetic background of HH and DP may be largely different, or shared by as yet undiscovered genes (Vaaralahti et al., 2011).

Loss-of-function mutations within the GnRH receptor are the most frequent cause of autosomal recessive CHH, accounting for 16%–40% of patients. Mutations have been found within the extracellular, transmembrane and intracellular domains of the receptor leading to impaired GnRH action (Chevrier et al., 2011). A homozygous partial loss-of-function mutation in *GNRHR* was found in two brothers, one with self-limited DP and one with idiopathic HH (Lin et al., 2006), and a further heterozygous mutation found in one male with self-limited DP (Vaaralahti et al., 2011).

### Genetic candidates for control of the pubertal ‘brake’

Our understanding of the reactivation of the gonadotropic axis at the end of the juvenile period, also seen as the release of the inhibitory ‘brake’ that has been restraining the HPG axis in childhood, remains incomplete. Puberty is marked by the change of the balance of GABA-glutamate signalling in the brain. This is associated with a higher dendritic spine density and a simplification of the dendritic architecture of GnRH neurons. GnRH neuronal activity is under the control of several neurotransmitters and neuropeptides, and the onset of puberty is triggered by a decline in these inhibitory signals and amplification of the excitatory inputs, leading to increased frequency and amplitude of GnRH pulses. However, the neuroendocrine mechanisms that act upstream to control and coordinate this activity remains unknown.

Kisspeptin, an excitatory neuropeptide, was identified as an instructive factor in puberty onset by the discovery of patients with GnRH deficiency with loss-of-function mutations in the *Kisspeptin 1 receptor*, *KISS1R* (previously known as *G-Protein Coupled Receptor 54, GPR54*) (de Roux et al., 2003; Seminara et al., 2003). Mice with knockout of *Kiss1r* were simultaneously discovered to be infertile despite anatomically normal GnRH neurons and normal hypothalamic GnRH levels (Seminara et al., 2003), with a phenotype consistent with normosmic GnRH deficiency. However, despite a large body of evidence for kisspeptin as one of the most important elements of the neural network responsible for GnRH pulse generation, only very rarely have human mutations in *Kisspeptin 1 (KISS1)* been found in patients with delayed or absent puberty (Topaloglu et al., 2012). Moreover, kisspeptin neurons in the arcuate nucleus have not been demonstrated as controllers of the release of the puberty brake, but instead are likely to act as a conduit for upstream regulators (Plant, 2015).

The excitatory neuropeptide, neurokinin b, also plays a role in the upstream control of GnRH secretion. Identification of this pathway was also via discovery of loss-of-function mutations in *TAC3*, encoding neurokinin b, and its receptor *TACR3*, in patients with normosmic GnRH deficiency and pubertal failure (Topaloglu et al., 2009). Kisspeptin, neurokinin b and dynorphin are coexpressed in KNDy neurons of the arcuate nucleus of the hypothalamus (de Croft et al., 2013), which project to and directly interact with GnRH neurons. Their expression is downregulated by oestrogen and testosterone as part of the negative feedback regulation of gonadotropin secretion (Rance, 2009; Dungan et al., 2006). Despite this, administration of neurokinin b agonists failed to stimulate GnRH release in rodents, and *Tacr3* knockout mice do achieve fertility when mated (Sandoval-Guzman and Rance, 2004; Kung et al., 2004). However, on closer phenotyping of *Tacr3* mice both males and females demonstrate central reproductive defects with potential for reversal of hypogonadism, highly reminiscent of the human phenotype (Yang et al., 2012). With respect to DP, in one study of 50 self-limited DP patients investigated for mutations in *TAC3* and *TAC3R*, only one mutation in a single patient was found in the latter gene (Tusset et al., 2012).

The inhibitory role of GABAergic neurotransmission has been clearly shown in primates (Mitsushima et al., 1994) but is more ambiguous in rodents. Opioid peptides provide additional inhibitory input but this appears to be less critical than the GABAergic signals in restraining the initiation of puberty (Ojeda et al., 2006). Additionally, *RFamide-related peptide* (*RFRP*), the mammalian ortholog of the avian peptide *gonadotrophin-inhibiting hormone* (*GnIH*), has been identified as a further inhibitory regulator of GnRH neuronal activity in mice (Ducret et al., 2009). Glial inputs appear to be predominantly facilitatory during puberty and consist of growth factors and small diffusible molecules, including TGFβ1, IGF-1 and neuregulins, that directly or indirectly stimulate GnRH secretion (Ojeda et al., 2008).

**Fig. 5:**
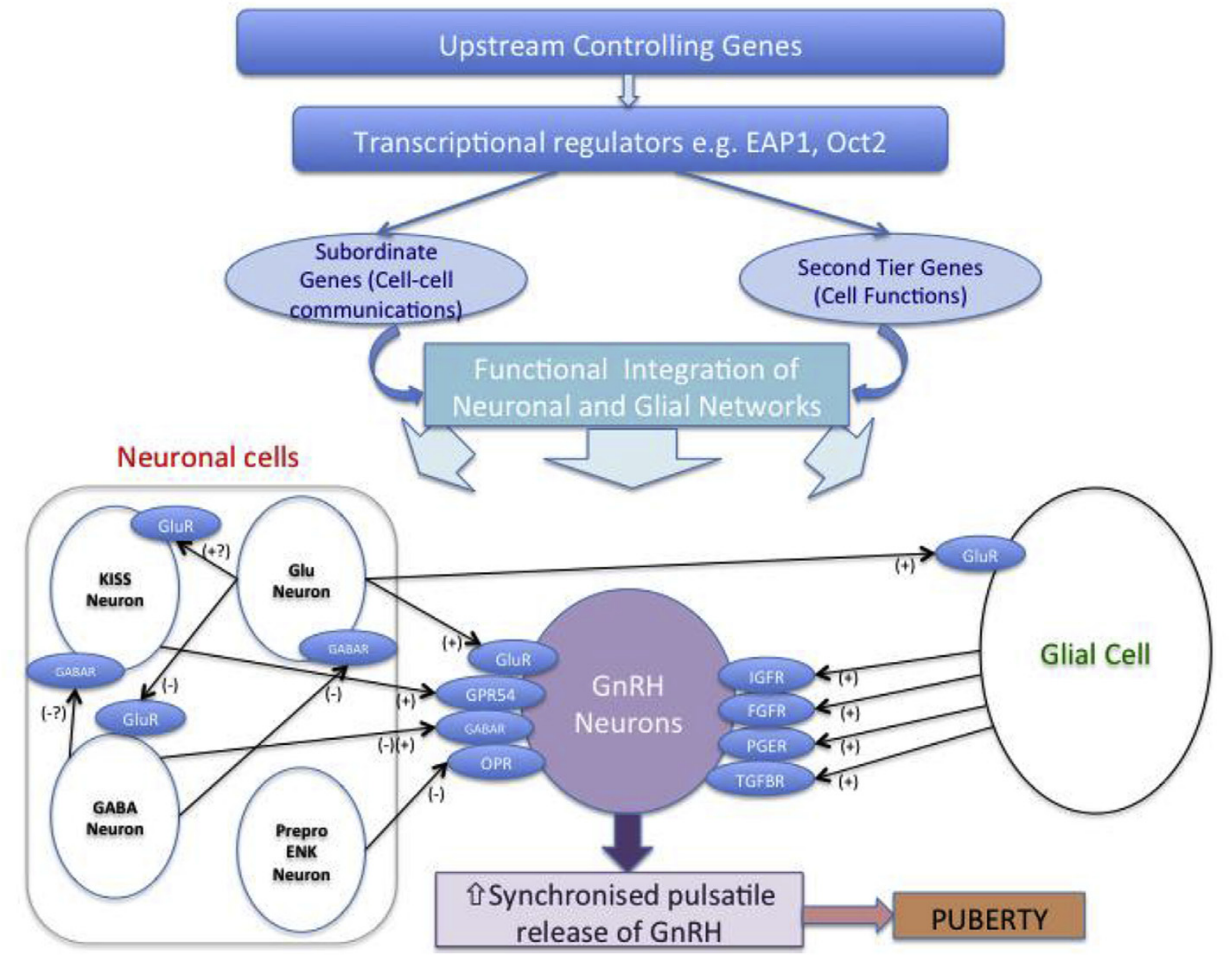
G**enetic regulators in the trans-synaptic and glial control of GnRH neurons during puberty, adapted from** (Ojeda et al., 2006). This schematic represents a model whereby key transcriptional regulators govern a plethora of other genes (termed “subordinate genes” and “second tier genes”, controlling cell-cell communications and cell functions respectively). This hierarchy, itself controlled by as yet unknown upstream controlling genes, integrates the neuronal and glial networks influencing GnRH neuronal function. Inhibitory inputs are primarily from GABAergic (GABA Neuron) and opiatergic neurons (preproenkephalinergic neurons, Prepro ENK), whilst glutamate (Glu neurons) and kisspeptin (KISS Neuron) are the central excitatory neuronal signals. Glial cell inputs are primarily facilitatory.

Upstream regulation of GnRH transcription is less well established. Candidate transcriptional regulators identified from a systems biology approach and animal models include *OCT-2*, *TTF*-1 and *EAP1* (Ojeda et al., 2010) (Fig. 5). *Oct-2* mRNA is upregulated in the hypothalamus in juvenile rodents, blockage of Oct-2 synthesis delays age at first ovulation whilst activation of Oct-2 expression (e.g. hamartomas) induces precocious puberty (Ojeda et al., 1999). *Ttf-1* (thyroid transcription factor-1) enhances GnRH expression, with increased expression in pubertal rhesus monkeys (Lee et al., 2001). *Eap1* mRNA levels also increase in the primate and rodent hypothalamus during puberty. Eap1 transactivates the GnRH promoter, and Eap1 knockdown with siRNA caused DP and disrupted estrous cyclicity in a rodent model (Heger et al., 2007). Recent data suggests Eap1 regulates GnRH expression independent of Kiss1 signalling (Li and Li, 2017). No published mutations in these upstream or regulatory factors have been reported in patients with DP. However, our group is completing functional annotation of two potentially pathogenic variants in *EAP1* found in our cohort of Finnish patients with self-limited DP (manuscript *in submission*).

Epigenetic regulators are potential modulators of pubertal timing. Recent evidence highlights the importance in mice of microRNAs (particularly the miR-200/429 family and miR-155) in the epigenetic up-regulation of GnRH transcription during the critical period (murine comparator of the mini-puberty) (Messina et al., 2016). Moreover, miR-7a2, has been demonstrated to be essential for normal pituitary development and HPG function, with deletion in mice leading to hypogonadotropic infertility (Ahmed et al., 2017). The effects of environmental changes on the hypothalamic regulation of puberty may be mediated in part via epigenetic mechanisms, and several studies have shown that the pubertal brain epigenome is affected by environmental perturbations. Moreover, maternal exposure to EDCs in rodents have been shown to cause epigenetic modifications in testis and other systemic effects, and thus epigenetic changes during foetal life are also a potential mechanism for the hypothalamic effects of EDCs in utero (Parent et al., 2015).

### 1.5 Pituitary genes controlling puberty

Downstream mutations in the GnRH signalling pathway can also present with DP. LH and FSH are glycoprotein hormones encoded by a common *α-*subunit gene and a specific *β-*subunit gene. Mutations of the *β-*subunits genes of LH or FSH are extremely rare causes of pubertal abnormalities (Themmen and Huhtaniemi, 2000). Males with inactivating mutations of the *LHB* have absent pubertal development with Leydig cell hypoplasia leading to T deficiency and azoospermia. Females with inactivating mutations of *LHB* present with onset of normal puberty, but with normal or late menarche followed by infertility due to lack of ovulation (Themmen and Huhtaniemi, 2000). Individuals with inactivating *FSHB* mutations present with incomplete pubertal development and azoospermia in males and primary amenorrhea in females (Layman et al., 1997).

Genetic defects affecting the development of the anterior pituitary may cause pubertal delay or failure. The pituitary transcription factors *LIM Homeobox 3(LHX3)*, *SRY-Box 2 (SOX2)* and *HESX homeobox 1 (HESX1)* are vital for early patterning of the forebrain and pituitary, and mutations in these developmental genes result in syndromic hypopituitarism with gonadotropin deficiency in humans (Kelberman et al., 2009). *Paired like homeodomain factor 1 (PROP1)* is important for the development of gonadotropin-secreting cells (Parks et al., 1999), and patients with *PROP1* mutations have variable GnRH deficiency ranging from DP to CHH (Kelberman et al., 2009). Mutations in *Orphan nuclear receptor Dax-1 (DAX1)* cause X-linked adrenal hypoplasia congenita with associated HH, but have not been found in isolated DP (Achermann et al., 1999).

Gonadotropin deficiency may also be associated with other conditions, particularly with neurological phenotypes. Mutations in *RNA polymerase III subunit A and B (POLR3A/B)* result in the 4H syndrome (Hypomyelination, Hypodontia and Hypogonadotropic Hypogonadism) (Wolf et al., 2014) whilst those in *Ring finger protein 216 (RNF216), OTU deubiquitinase 4 (OTUD4)* and *Patatin like phospholipase domain containing 6 (PNPLA6)* produce the phenotypic combination of HH and ataxia (also known as Gordon-Holmes syndrome) (Margolin et al., 2013; Topaloglu et al., 2014). *DMX like 2 (DMXL2)* mutations are associated with congenital HH, other endocrine deficiencies and polyneuropathies (Tata et al., 2014). Dysregulation of the RAB3 cycle, such as with mutations in *RAB3 GTPase activating protein catalytic subunit 1 (RAB3GAP1)*, lead to Warburg Micro syndrome with ocular, neurodevelopmental and central reproductive defects (Aligianis et al., 2005; Bem et al., 2011).

### 1.6 Delayed puberty due to primary gonadal failure

In gonadal dysgenesis in both males and females, delayed or absent pubertal development may be the presenting complaint, although associated features usually predominate. In Turner syndrome, the most common form of hypergonadotropic hypogonadism in females, puberty is delayed and usually followed by progressive ovarian failure (Saenger et al., 2001). Importantly, however, up to 30% of girls will undergo spontaneous pubertal development and 2–5% will have spontaneous menses (Improda et al., 2012). About half of girls with Turner syndrome have the 45,X karyotype. Other causes of ovarian dysgenesis include X isochromosome, where abnormal chromosome division results in duplication of identical chromosome arms, most commonly of the long (q) arm. Various deletions and duplications of the short and long arm of the X chromosome are also found in women with primary ovarian insufficiency, with several genes implicated including *Fragile X mental retardation 1 (FMR1), Premature ovarian failure 1B (POF1B)*, *Diaphanous related formin 2 (DIAPH2)*, *Forkhead box L2 (FOXL2)* and *Bone morphogenetic protein 15 (BMP15)* (Cox and Liu, 2014). Point mutations in the extra-cellular domain of the FSH receptor are mostly restricted to the Finnish population and result in inactivation of the receptor function with primary or secondary amenorrhea (Aittomaki et al., 1995).

In males, testicular abnormalities are characterized by elevated gonadotropin and low inhibin-B concentrations, and may present as pubertal delay. The commonest condition underlying hypergonadotropic hypogonadism in males is Klinefelter syndrome (47,XXY), with a prevalence of 1 in 667 live births. The majority of those affected will enter puberty spontaneously at a normal age (Juul et al., 2011), but testosterone levels become increasingly deficient by Tanner stages 4–5, possibly as a result of secondary regression. DP may be seen in those with a more complex karyotype (48,XXYY, 48,XXXY, 49,XXXXY).

### 1.7 Genetics of pubertal timing in the general population

Over the last decade there have been several large genome wide association studies (GWAS) of age-at-menarche, examining pubertal timing in healthy females, and more latterly also in males (Ong et al., 2009; Perry et al., 2014; Elks et al., 2010). These studies have sought to identify key genetic regulators of the timing of puberty in humans and have demonstrated that there is significant genetic heterogeneity in pubertal timing in the general population. These data suggest that the genetic architecture of the timing of puberty in healthy subjects is likely to involve at least hundreds of common variants. The first of many loci associated with age of menarche was the gene *Lin-28 homolog B (LIN28B)* (Perry et al., 2009). *LIN28B* is a human ortholog of the gene that controls, through microRNAs, developmental timing in the *Caenorhabditis elegans*. However, mutations in *LIN28B* have not to date been identified in human patients with DP (Tommiska et al., 2010) or precocious puberty (Silveira-Neto et al., 2012).

The largest study of this type comprises 1000 Genomes Project-imputed genotype data in up to ∼370,000 women, and identifies 389 independent signals (P < 5 × 10^−8^) for age at menarche (Day et al., 2017). Per-allele effect sizes ranged from 1 week to five months. These signals explain ∼7.4% of the population variance in age at menarche, corresponding to ∼25% of the estimated heritability. Many of these signals have concordant effects on the age at voice breaking, a corresponding milestone in males. However, in women the signals identified had stronger effects on early than on late age of menarche, but in contrast had larger effect estimates for relatively late than relatively early voice breaking in males (Day et al., 2017).

Around 250 genes were identified via coding variation or associated expression, particularly those expressed in neural tissues. Importantly, genes already implicated in rare disorders of puberty were identified, including *Leptin receptor (LEPR)*, *Gonadotropin releasing hormone 1 (GNRH1)*, *KISS1* and *TACR3*, and signals in or near several further genes with relevance to pituitary development and function including *POU Class 1 Homeobox 1(POU1F1)*, *Teneurin Transmembrane Protein 2 (TENM2)* and *Leucine Rich Repeat Containing G Protein-Coupled Receptor 4 (LGR4).* Two imprinted genes were also reported: *Makorin ring finger protein 3 (MKRN3)*, paternally-inherited mutations in which have been identified as causal in pedigrees of central precocious puberty (CPP) (Abreu et al., 2013); and *Delta Like Non-Canonical Notch Ligand 1 (DLK1)* (Dauber et al., 2017). *MKRN3* is thought to contribute to the puberty ‘brake’ restraining the HPG axis via inhibition of GnRH release. However, neither *MKRN3* nor *DLK1* mutations have been described in the pathogenesis of DP (Fig. 4).

### 1.8 Metabolism and timing of puberty

Nutritional changes play an important role in the observed secular trend towards an earlier age of pubertal onset in the developed world (Ong et al., 2006), as shown by the positive correlation between age at puberty onset and childhood body size, particularly in girls (Biro et al., 2006). In contrast, under-nutrition in females, for example in chronic disease or anorexia nervosa, can result in delay in both the onset and tempo of puberty (Frisch and McArthur, 1974).

This relationship between fat mass and pubertal timing is partly mediated through the permissive actions of the metabolic hormone leptin, a key regulator of body mass, produced from white adipose tissue (Elias, 2012). Humans and mice lacking leptin (Lep ob/ob) or the leptin receptor (LepR db/db) fail to complete puberty and are infertile (Barash et al., 1996). However, whilst self-limited DP in boys is associated with hypoleptinaemia (Gill et al., 1999), there have been no identified association of specific leptin or leptin receptor polymorphisms with DP (Banerjee et al., 2006). GWAS studies of pubertal timing found, in addition to leptin signalling, overlap with several genes implicated in body mass index including *Fat mass and obesity-associated protein (FTO)*, *SEC16 homolog B (SEC16B)*, *Transmembrane protein 18 (TMEM18)*, and *Neuronal growth regulator 1 (NEGR1)* (Day et al., 2017).

Very recently, rare heterozygous variants in *FTO* have been identified in pedigrees with self-limited DP associated with extreme low BMI and maturational delay in growth in early childhood (Howard et al., ). Notably, mice that are heterozygous for *FTO* gene knockout displayed significantly delayed timing of puberty, without significant reduction in body mass. FTO is known to function as a RNA demethylase linking amino acid availability, via mTOR, to appropriate levels of growth and translation (Speakman, 2015), although may also act in concert with other genes in the nearby region to exert effects on body weight. There is evidence that mTOR plays a central role in the coupling of energy balance and HPG axis activation, via modulation of hypothalamic expression of Kiss1 (Roa et al., 2009; Martinez de Morentin et al., 2014). Blockade of mTOR caused delayed vaginal opening in rodents with blunting of the positive effects of leptin on puberty onset in food-restricted females. It remains to be determined if the effect of *FTO* on pubertal timing in self-limited DP is mediated via effects on body mass, via mTOR signalling, or both.

*α*-MSH signalling via MC3/4 receptors, acting to increase Kiss1 expression and mediate the permissive effects of leptin on puberty, has also been implicated recently as an important element in the metabolic control of puberty (Manfredi-Lozano et al., 2016). Ghrelin and other gut-derived peptides may also form part of the mechanism by which energy homeostasis regulates reproductive development (Pomerants et al., 2006; Fernandez-Fernandez et al., 2006). A small cohort of 31 patients was analysed for mutations in the ghrelin receptor *Growth Hormone Secretagogue Receptor (GHSR)* and 5 patients were found to have point mutations in this gene (Pugliese-Pires et al., 2011).

Children with CDGP have a dual phenotype of slow growth in childhood with DP. In contrast, both low birth weight and prematurity are associated with earlier onset of puberty (Persson et al., 1999), particularly in those children with rapid increase in length or weight in the first two years of life (Wehkalampi et al., 2011). It is not clear, however, if childhood obesity, insulin resistance, excess androgens or underlying genetic or epigenetic factors may explain this association (Dunger et al., 2006).

## 2 Conclusions

The mystery of which are the key controllers of the duration of dormancy of the HPG axis after the mini-puberty, and what triggers the release of this puberty ‘brake’, has yet to be answered. A wide variety of genetic and epigenetic defects affecting different aspects of the HPG axis at different time periods in fetal and postnatal life may result in delayed and disordered puberty. Whilst familial self-limited in DP is a highly heritable trait with evidence for a genetic basis, the majority of these genes remain unknown. Although our understanding of the highly complex underlying biological network remains imperfect, results to date demonstrate the importance of defects in GnRH neuronal development and function, GnRH receptor and LH/FSH abnormalities, transcriptional regulation of the HPG axis and metabolic and energy homeostasis derangements in the pathogenesis of self-limited DP. This review serves to highlight the high degree of heterogeneity in the genetic basis of self-limited DP.

Clinically it is important to distinguish between the conditions of DP and idiopathic CHH in adolescents presenting with DP. However, this diagnosis is often a difficult one as both disorders can present with a picture of functional hypogonadism. There is still no definitive test to accurately discriminate between the two diagnoses. More complex and involved management is required in patients with CHH to achieve both development of secondary sexual characteristics and to maximize the potential for fertility (Boehm et al., 2015). Genetic testing may inform diagnosis of associated syndromic features, likelihood of reversal and inheritance in family members. Rapid and efficient diagnosis of patients in clinic would represent a huge leap forward in patient care and a likely significant economic advantage. While presently next generation sequencing in individuals presenting with delayed or incomplete pubertal development is only a reasonable option in a research setting, future progress in gene discovery and technical developments may facilitate the availability of genetic diagnosis as part of clinical care for patients with both GnRH deficiency and self-limiting DP.
